# Effects of active video games on physical activity among overweight and obese college students: a systematic review

**DOI:** 10.3389/fpubh.2024.1320112

**Published:** 2024-02-13

**Authors:** Yue Zhao, Kim Geok Soh, Hazizi Abu Saad, Chunqing Liu, Cong Ding

**Affiliations:** ^1^Faculty of Educational Studies, Department of Sports Studies, Universiti Putra Malaysia, Selangor, Malaysia; ^2^Faculty of Medicine and Health Sciences, Department of Nutrition, Universiti Putra Malaysia, Selangor, Malaysia

**Keywords:** exergame, moderate-to-vigorous physical activity, energy expenditure, obesity, young adult

## Abstract

**Background:**

The purpose of this study was to critically review the existing literature on the effects of active video games (AVGs) on physical activity in overweight and obese college students to determine whether AVGs can promote and achieve recommended levels of physical activity. The results should provide constructive input for future research.

**Methods:**

A total of five international databases including PubMed, SCOPUS, Web of Science, CINAHL Plus, and EBSCOhost were searched with keywords related to “active video games,” “physical activity,” and “obese college students” from July 2022. A systematic review was conducted using the PRISMA guidelines and randomised controlled trial (RCT) studies that met the inclusion criteria were included. Furthermore, the quality assessment of the studies was measured using the PEDro scale.

**Results:**

One thousand and twenty-three articles were retrieved, of which eight randomised controlled trial studies met the inclusion criteria. AVGs can reduce sedentary behaviour and positively affect physical activity, time spent on moderate-to-vigorous physical activity (MVPA), positive psychological factors, and game attendance rate. Combining AVGs with other assistive devices (such as mini-trampolines and stationary bikes) can enhance the effects of AVGs and provide greater physiological stimulation. Different types of AVGs and game modes can achieve different emotional responses, physiological stimulation, and physical activity levels.

**Conclusion:**

The research findings prove that AVGs can be a viable intervention to increase physical activity in overweight or obese college students, ultimately reaching the recommended physical activity level(PAL). Physical activity can be further increased by incorporating assistive devices or using features supported by self-determination theory (SDT). As a new modality, AVGs could be a potential alternative to traditional physical activity.

**Systematic Review Registration:**https://www.crd.york.ac.uk/prospero, identifier: CRD42022363993.

## 1 Introduction

Obesity is a severe health issue affecting individuals across all age cohorts in developed and developing nations (1, 2). Being overweight entails having a body mass index (BMI) greater than 25 kg/m^2^, while obesity entails an index greater than 30 kg/m^2^ (3). Overweight and obesity can cause a variety of diseases, such as cardiovascular disease and hypertension (4, 5). Many factors contribute to overweight and obesity such as diet, genetics and environment (6). However, with the advancement of technology, people are becoming more connected to digital devices, their lifestyles are gradually moving from offline to online and they spend a lot of time in the digital world every day (7, 8). Many studies have shown that over-reliance on digital (e.g., use of mobile phones, tablets, and computers) has become a new way of becoming overweight and obese (9, 10).

We are currently experiencing a surge in physical inactivity and sedentary behaviour. According to The Lancet, 81% of adolescents and 27.5% of adults globally have insufficient physical activity and WHO’s 2025 global target of reducing the number of physically inactive people by 10% is difficult to achieve (11, 12). Insufficient physical activity combined with prolonged sedentary life can easily lead to obesity (13). Obesity has become more common among college students and can result in poor physical health, discrimination, low self-efficacy, and depression (14). A person’s college years largely determine physical activity, eating habits, and behaviour. Effective interventions during this period can prevent long-term adverse health outcomes (15). The American College of Sports Medicine (ACSM) and the Centres for Disease Control and Prevention (CDC) have formulated different physical activity guidelines. According to these guidelines, adults should exercise 75 min a week vigorously or 150 min moderately and use 150 kilocalories (kcal) per day and 1,000 kcal per week for optimal health (16). Research has concluded that moderate physical activity can reduce digital addiction and obesity (17) and experts suggest many ways to achieve the recommended physical activity level through traditional physical activities like cycling and walking. However, many college students prefer to avoid these conventional physical activities (18). Therefore, an essential requirement exists to encourage physical activity among overweight and obese college students through alternative exercise modalities, such as AVGs.

The progress of information technology and the popularity of electronic devices have increased the frequency of computer and cell phone use among college students (19). Overweight and obese college students are a particular group of college students. They are accompanied by abdominal or hip obesity, are often sedentary or physically inactive (less physical activity than recommended by the World Health Organisation), and are very fond of playing video games. Taking advantage of overweight and obese college students’ interest in screen games, converting static screen time to dynamic screen time and getting them moving while using the screen would be a new way to control sedentary behaviour and obesity. An AVG is also known as an “exergame”; it requires players to interact with images on the screen through upper-body, lower-body, and whole-body movements to complete the game (20). Traditional video games operate by fingers only and are associated with sedentary behaviour. AVGs differ from conventional static screen games (21). They combine screen, game, and participant through multiple game controllers to motivate participants to keep moving and thus improve physical activity (22).

Numerous types of research have demonstrated that AVGs can elicit more significant EE and elevate PALs in contrast to sedentary video games (static games). AVGs may meet moderate-to-vigorous physical activity (MVPA) requirements (23). AVGs also improve oxygen uptake, heart rate, and physical fitness while eliciting higher enjoyment and psychological well-being (24). The exercise effects of different types of AVGs may vary, with some studies reporting that both aerobic and strength activities in Nintendo Wii Fit can achieve moderate physical activity and serve as a new tool for improving physical activity among college students (25). Other studies suggest that exergames, such as boxing and soccer in Xbox 360 Kinect, can significantly increase vigorous physical activity more than playing ping pong or bowling (26). Additionally, AVGs are a viable alternative to traditional physical activity during school lunch breaks for elementary school students and can thus help achieve the physical activity intensity recommended by physical activity guidelines (27). Regarding weight loss, AVGs may increase activity levels among obese adolescents and serve as a potential tool for treating obesity (28).

Active video games in general have grown to become one of the most popular forms of entertainment in the world (29), not only promoting the integration of recreation and health (30) but also having the potential to reduce the risk of premature death and cardiovascular disease and to improve overall health and well-being. Numerous pieces of literature prove the health benefits of AVGs and can lead to an active lifestyle, becoming a valuable new way to promote public health (31–34). However, much of the current research focuses on applying AVGs to physical activity promotion in children and adolescents (35–37), rehabilitation in older adults (38) and special populations such as Alzheimer’s (39) and adjunctive therapy for autism (40). Overweight and obese college students, as young adults, spend a lot of time on digital games, often accompanied by sedentary behaviour, digital addiction and lack of physical activity (41, 42). However, the current relationship between interventions for physical activity using AVGs remains unclear. Therefore, this systematic review endeavours to determine the effects of AVGs on physical activity in overweight and obese college students, relying on the most recent evidence from randomised controlled trials (RCTs).

## 2 Methods

### 2.1 Protocol and registration

The systematic review followed the Preferred Reporting Items for Systematic Reviews and Meta-Analyses (PRISMA) guidelines for protocol selection, data selection, collection, and analysis (43). It complied with ethical standards in sports and exercise science research (44). The systematic review was registered in the international prospective systematic review database (registration number: CRD42022363993).^1^

### 2.2 Data sources and search strategy

The literature was searched through five well-known international databases: PubMed, Web of Science, EBSCOhost (SPORTDiscus), CINAHL Plus, and Scopus. Search scope covers until the end of 2022. The investigation was conducted by title in each database, and the following predefined keywords, combinations of keywords, and Boolean operators “and” and “or” were utilised. The search strategy was “video gam*” OR “videogam*” OR “active video gam*” OR “exergam*” OR “interactive gam*” OR “interactive video gam*” OR “exercise video gam*” OR “fitness gam*” OR “Playstation” OR “Nintendo” OR “Wii” OR “Xbox” OR “Kinect” OR “EyeToy” AND “physical activity” OR “energy expenditure” OR “exercise” OR “energy metabolism” OR “bodily movement” OR “heart rate” OR “metabolic equivalent” OR “oxygen consumption” OR “activity count*” AND “obes*” OR “overweight” AND “college student*” OR “university student*” OR “undergraduate student*” OR “graduate student*.” Furthermore, searches were conducted in two ways for additional supplementation to retrieve the publications as comprehensively as possible. The first method was entering keyword combinations into Google Scholar to obtain additional literature. The second was checking reference lists from previous relevant articles and meta-analyses and manually filtering and sifting through the literature titles for possible matches.

### 2.3 Eligibility criteria

The patients, intervention, comparison, outcomes, and study design (PICOS) principle was employed to identify the articles included in this study. Each factor in PICOS formed a category in this study and formed an inclusion criterion. The titles and abstracts of all retrieved articles were subjected to relevance screening. Then the full texts of eligible studies were obtained and appraised based on the predetermined inclusion criteria, as presented in Table 1. Consequently, studies were incorporated into the review only if they fulfilled the specified inclusion criteria. This systematic review included only randomised controlled trials, which can better explain the cause-and-effect relationship between active video games and physical activity than other studies.

- The study participants are college students without disease and with a BMI that meets the requirements for overweight or obesity without differentiating between their age, and gender.
- AVGs are utilised as an intervention and do not differentiate between usage sites.
- Comparisons in a study should include at least one experimental group and a control group.
- Study results comprise at least one assessment of the effect of AVGs on physical activity.
- The experimental design is an RCT.

**Table 1:** PICOS eligibility criteria.

PICOS	Detailed information
Population	Overweight and obese college students
Intervention	Active video games
Comparison	Two or more groups’ trials
Outcome	Objective physical activity outcomes (such as EE, METs, RPE, HR, and VO_2_) and physical activity level (such as MVPA time and percentage of time in MVPA)
Study design	Randomised controlled trials

On the other hand, studies that met the following exclusion criteria were excluded:

- Articles are not published in English.
- Publish in a peer-reviewed journal by December 2022.
- Experimental studies that are not about AVGs or physical activity.

### 2.4 Study selection

Firstly, searches were conducted through five internationally accessible databases, and all the obtained studies were imported into Zotero software, which can achieve literature management. The software’s de-duplication functionality removed duplicates. Secondly, two independent reviewers (Liu and Ding) primarily screened the de-duplicated literature. After downloading the literature, they screened it according to the established criteria to determine which articles to include. Both independent reviewers (Liu and Ding) worked separately. When there was a disagreement about which article to select, an independent third-party reviewer (Soh) would conduct a comprehensive analysis of the article in question and continue to evaluate it until they reached a consensus.

### 2.5 Data extraction and quality assessment

After screening the studies, the following essential data were obtained from eligible studies in a pre-formulated extraction format: 1. Authors’ names, titles, and years of publication; 2. Sample characteristics; 3. Intervention characteristics (type, indicators, frequency, and duration); 4. Study design; and 5. Research results.

The Physiotherapy Evidence Database (PEDro) is a scale for assessing the methodological quality of trials. The total PEDro score is valid as an indicator of methodological quality (45). It is also reliable for systematically evaluating methodological quality with high reliability (46). The scale comprises 11 items, and the scoring system is “no” (0 points) and “yes” (1 point). The final score of each study was obtained by summing the scores of these 11 items and higher PEDro scores represent higher methodological quality. The following criteria were used: a PEDro score < 6 indicates “low quality/high-risk bias,” and a score ≥ 6 indicates “high quality/low-risk bias.”

## 3 Results

### 3.1 Study selection

**Figure 1:**
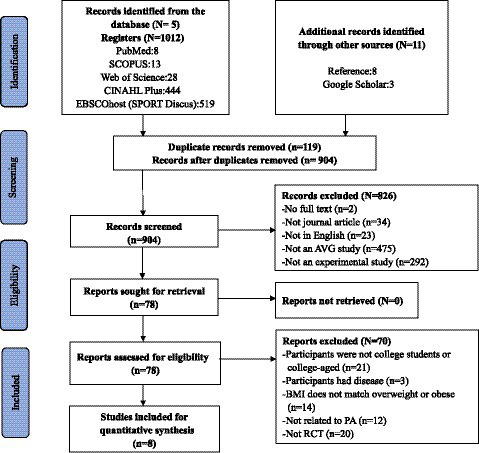
PRISMA flow chart of study selection.

Figure 1 illustrates the record selection process of searching through electronic databases. Potential studies for 1023 were identified. Five internationally recognised databases provided 1,012 of these 1,023 studies (8 from PubMed, 13 from SCOPUS, 28 from Web of Science, 519 from EBSCOhost (SPORT Discus), and 444 from CINAHL Plus). Other sources provided the other 11 studies (3 from Google Scholar and 9 from Reference). After removing 119 duplicates, 904 articles were screened and evaluated individually. After removing 826 pieces by subject and abstract, the remaining 78 articles were read according to the inclusion criteria developed, of which 70 did not match. Therefore, eight RCT reports were finally included to evaluate the effect of AVGs on physical activity among overweight and obese college students. The publication dates of these eight articles range from 2007 (47) to 2019 (48) and include one from Indonesia (48), one from Canada (47), two from Brazil (49, 50), and the rest from the United States.

### 3.2 Study quality assessment

Table 2 demonstrates the scoring information of the PEDro scale included in the literature. The eight included articles’ PEDro scores ranged from 4 to 7 points (mode = 6; mean = 5.6; median = 6). Specifically, the score of the PEDro scale in three studies was less than 6, and the score of the other five studies was greater than or equal to 6. All studies were deducted points related to blindness in participants, assessors, and therapists. Since the included studies used AVGs for intervention, requiring field testing and field instruction, it is difficult to blind participants, assessors, and therapists.

**Table 2:** Scores of the methodological quality assessment.

References	Jacobs et al. (51)	Peng et al. (52)	Warburton et al. (47)	Rodrigues et al. (50)	Rahayu et al. (48)	Russell et al. (53)	Douris et al. (54)	De Brito-Gomes et al. (49)
Eligibility criteria	1	1	1	1	0	1	1	1
Random allocation	1	1	1	1	1	1	1	1
Allocation concealment	0	0	0	0	0	0	0	0
Baseline comparability	1	1	1	1	1	1	1	1
Blind participants	0	0	0	0	0	0	0	0
Blind therapist	0	0	0	0	0	0	0	0
Blind assessor	0	1	0	0	0	0	0	0
Follow-up	1	1	1	1	1	0	1	1
Intention to treat analysis	0	0	0	0	0	0	0	0
Between group comparisons	0	1	1	1	1	1	1	1
Point measure and variability	0	1	1	1	1	1	1	1
Total PEDro score	4	7	6	6	5	5	6	6

### 3.3 Population characteristics

Table 3 illustrates the population characteristics from the following aspects:

1. Nationality: Only one of the included studies presented the nationality of one of the participants as Mexican (51).
2. Ethnicity: Two studies reported information about ethnicity, including 92 whites, 13 Asians, 12 African Americans, and nine other races (52).
3. Gender: One study had only female participants (51). Four studies had only male college students (47–50). The total number of participants was 234 participants, including 108 males and 36 females, and the remaining 90 participants did not report gender (52).
4. Age: Five studies reported an age range greater than 18 years (51), four of which ranged from 18 to 25 years (47, 49, 52, 54).
5. Sample size: There were 234 subjects in these eight studies, with participants ranging from 5 to 121 (51, 52). The median was 20, and the mean was 29.25.
6. BMI: Six studies described BMI (47–49, 51, 52, 54).
7. Educational background: Two studies indicated that the subjects were college-aged (47, 53) while the other six were college students.

**Table 3:** Characteristics of included studies.

Authors (Year) Country	Study description	Population	Frequency and duration	AVGs	Instrument	Measurement and main outcome	Key findings
Jacobs et al. (51)	To determine the feasibility of Nintendo Wii Fit to encourage undergraduate students to lose weight through a 12-week intervention program.	*F* = 5 Age ≥ 18 BMI ≥ 18 Race: Asian(n = 2,40%). White (n = 2, 40%). Mexican (n = 1, 20%).	Experimental group: 45 min each time, 4 times a week. Control group: walking to class. Duration is 12 weeks.	Nintendo Wii Wii Fit	1.Pedometer 2.Standard bathroom scale or Wii Fit balance board 3.Activity Level Test Questionnaire 4.BREQ-2 5.PAR-Q	1.Body weight and BMI. 2.Participants’ current activity level, personal exercise motivation, and physical health. 3.Measure RPE every Wii session. 4.Record steps, activity and self-reported daily food every day BMI↓, weight↓ sports motivation↑, PAL ↔	Using Wii Fit for exercise can increase exercise motivation and be used as a tool for college students to control their weight individually.
Peng et al. (52)	To assess the potential of active video games informed by theoretical guidance to enhance both MVPA and game involvement among college students, while also decreasing their sedentary behaviour.	*F* = 56; M = 65 (Invalid number, n = 31) Age: 18–25 Race: Asian (n = 11, 9.1%). White(n = 90,74.4%). African American (n = 12, 9.9%). Other (n = 8, 6.6%).	Experimental group: Three times a week for 4 weeks. Control group: no treatment.	A third person, fantasy role-playing game	ACTi Graph GT3X accelerometers PACES Active inputs (Wii mote, Nunchuk, and dance pad) Controller input (Xbox 360 controller)	1.Baseline measurement in week 0 2.Physical activity was objectively measured at week 0 (baseline), and week 1 and 5. 3.Calculate the attendance rate 4.Measure the players’ enjoyment, perceived need satisfaction of competence, and future play motivation in the first and fourth weeks. MVPA↑, attendance↑, psychological need satisfaction↑	An AVG designed with the motivation theory has a positive impact on attendance and MVPA, and can improve the effectiveness of AVG.
Warburton et al. (47)	To assess the efficacy of AVGs (combined with stationary cycling) in enhancing exercise adherence relative and physical fitness when compared to cycling training.	M = 14 Age: 18–25 BMI: Intervention group =27 ± 6. Control group = 28 ± 6.	30 min each time, three times a week. Duration is 6 weeks.	Sony PlayStation Game Bike	1.Polar s-510 2.Bioelectrical impedance 3.Television monitor 4.Electronically braked cycle ergometer 5.Metabolic cart 6.CPALFA 7.Vertec device 8.Flexometer 9.Pulse Wave	1.Attendance rates were monitored. 2.Physical fitness (VO2max, body composition, muscle strength, muscle burst, and flexibility) and resting blood pressure. VO_2_max↑, BM↔, BMI↔, FM↔, LBM↔, RSP↓, SBP↓	Compared to traditional bicycle training, AVG combined with fixed bicycles can lead to greater health improvements, and higher attendance leads to more physical activity.
Rodrigues et al. (50)	To compare metabolic and cardiovascular responses when using the AVG with and without the Mini trampoline.	M = 19 Age: 20.6 ± 2.01	Eight minutes each time, two times in total (with an interval of 2 days). Duration is 1 week.	Nintendo Wii Fit Plus Free Run	Bioelectrical Impedance Analyzer VO2000 portable automated system, treadmill, and prevent Pneumotach Polar	Day 1: measure height, weight, body composition, VO_2_ max and HR max Day 4: VO_2_, HR, and METs were measured in both MT-PT and EXG-PT groups. Day 7: experimental and control groups exchange game conditions. PAL↑, %VO_2_max↑, %HRmax↑, METs↑	Playing AVG on MT has greater physiological needs than playing AVG in traditional ways and can achieve moderate to intense physical activity. MT is an additional tool to enhance the physiological response when playing AVG.
Rahayu et al. (48)	To determine the effect of playing AVG (only use the upper and lower limbs) on BG, HR, and SpO2.	M = 24 Age: ±20 BMI: upperEx =24.6 ± 2.93. lowerEx =23.4 ± 2.47. control =24.6 ± 3.15.	Play avg. of 30 min. Lasts 120 min.	XBOX or Nintendo Wii	1.Accucheck Performa glucometer 2.OxyOne Finger Pulse Oximeter	1.Measure fasting BG, HR, SpO2 2.A sweet drink was given to all participants. Measure BG, HR, SpO2 at 30, 60, 90, 120 min 3.AVG groups played AVG within 30 to 60 min, while the control group did not exercise. SpO2↔, HR↔,BG↓	Only the upper and lower AVGs effect BG and HR, but will not affect the SpO+ of college students. Playing with upper and lower limb AVG is beneficial for health.
Russell et al. (53)	To examine AVG’s physiological and affective responses across user experience and social conditions (paired vs. solo).	*F* = 19; M = 29 Age: *F* = 21.4 ± 5.6. M = 22.4 ± 5.7.	30 min each time, two times total (Paired mode and solo mode).	Nintendo Wii Punchout	1.PAR-Q 2.Polar 3.Body fat analyser 4.StepWatch 3 pedometers 5.Borg’s modified 10-point scale 6.POMS	1.Physical Measures: in solo and paired mode, record heart rate every minute, and measure RPE every 5 min. Measure RecHR and SeRPE for 15 min after exercise. 2.Mood: Use the POMS scale 5 min before exercise, 5 min and 15 min after exercise to measure mood changes. resting HR↔, Steps↔, mood↔	Physical and emotional responses are not influenced by experience or social conditions when playing Wii punchout. Using AVG as a potential alternative to traditional sports activities requires consideration.
Douris et al. (54)	To contrast the physiological and psychological reactions between college students engaging in Nintendo Wii Fit games and those participating in moderate-intensity brisk walking for an equivalent duration.	*F* = 12; M = 9 Age: 23.2 ± 1.8 BMI:23.7 ± 3.7	30 min per day for 2 days. (One week apart).	Nintendo Wii Fit Free Run	1.PAR-Q 2.Standing blood pressure cuff 3.Stethoscope 4.Digital body weight scale, stopwatch, and measuring tape. 5.SEES 6.Treadmill	1.Every session was measured at 5-min intervals for HR, RR, BP, and RPE. 2.After each exercise condition, measurements of HR, BP, RPP, RPE, and SEES were recorded. HRmax↑, RPP↑, RPE↑, positive well-being↓	Wii Fit Free Run could serve as a viable alternative to conventional moderate-intensity aerobic exercise.
De Brito-Gomes et al. (49)	To examine and contrast the effects of structured and unstructured AVG on physical activity and motivation. Use indirect measure analysis (heart rate) to verify the effect of AVG on physical activity.	M = 13 Age: 19.4 ± 1.6 BMI: structured AVG = 23.6 ± 3.3. unstructured AVG = 22.6 ± 2.0	30 min each time, three times a week. Duration is 6 weeks (18 lessons).	XBOX 360 Kinect Kinect Sports: Boxing 2. Nike Kinect: Training	PAR-Q Polar FT1 2.Automated blood pressure machine 3.Metabolic gas analyser 4.Borg Scale (6–20 points) 5.Multimedia projector	1.Baseline assessments of height, BW, resting HR, BP, AE (VO2max), and peak workload. 2.Hemodynamic and inotropic measures SBP and DBP 3.In each session, measure maximum HR and average HR, RPE, EE, and MET through mathematical formulas. AE↔, pkHR↔,RPE↔, motivation↔, averge HR↑, MET↑, EE↑	The indirect measurements can analyse physical effort intensity. Structured AVG shows better HR and EE response than unstructured AVG.

BREQ-2, the Behavioural Regulation in Exercise Questionnaire-2; PAR-Q, Physical Activity Readiness Questionnaire; F, female; M, male; RPE, ratings of perceived exertion; SDT, self-determination theory; PACES, The Physical Activity Enjoyment Scale; CPAFLA, Canadian Physical Activity Fitness and Lifestyle Approach; MT, mini-trampoline; MT-PT, play AVG on mini-trampoline; EXG-PT, play AVG on wooden surface; BG, blood glucose; HR, heart rate; OSL, oxygen saturation levels; SpO_2_, the percentage of oxygen saturation; upperEx, upper only extremities exergaming; lowerEx, only extremities exergaming; HRR, heart rate reserve; THR, target heart rate; pkHR, peak heart rate; RecHR, recovery heart rate; SeRPE, post exercise session rating of perceived exertion; POMS, The Profile of Mood States; RPP, rate pressure product; BP, blood pressure; BW, body weight; RSP, resting blood pressure; RR, respiratory rate; SEES, Subjective Exercise Experience Scale; SBP, systolic blood pressure; DBP, diastolic blood pressure; EE, energy expenditure; MET, metabolic equivalent; BM,body mass; BMI, body mass index; FM, fat mass; LBM, lean body mass;↑, significant improvement; ↓, significant decrease; ↔, no significant difference.

### 3.4 Intervention characteristics

Table 3 demonstrates several underlying components of the intervention characteristics in the included studies, mainly including the following.

1. Intervention type: Two studies employed AVGs in combination with other devices (bicycles and mini-trampolines) (47, 50).The remaining six utilised AVGs directly as the intervention (48, 49, 51–54).
2. Intervention duration: A study did not specify the duration, only on two different days (53). The remaining studies ranged from 1 day to 12 weeks (48, 51). Two of the studies were 6 weeks (47, 49) and two studies were 1 week (50, 54). One study was 4 weeks (52). As for each AVG session, only one study did not report the duration of each session (52). Four studies had 30-min intervention sessions (47, 49, 53, 54), and the remaining two studies had 45-min and 8-min sessions, respectively (50, 51).
3. Intervention frequency: One study did not report the frequency of intervention (53), and one study conducted only one intervention (48). The frequency of intervention in the remaining six studies ranged from two to four times a week.
4. Type of AVG: Four studies utilised the AVGs of Wii produced by Nintendo (50, 51, 53, 54). The remaining studies utilised Microsoft’s Xbox (49), Sony’s PlayStation 2 (47), and self-designed third-person role-playing game (52). One study utilised Xbox and Wii (48).
5. Control groups: No intervention for the control group in the three studies (48, 51, 52), and the control groups of the remaining five studies used AVGs under different conditions.

### 3.5 Outcome and measures

*Physical activity* is any bodily movement generated by skeletal muscles that results in energy expenditure (55). The following physical activity level (PAL) categories provide a better understanding of the effect of AVGs on physical activity: (1) ACSM and CDC classify PAL into three categories: light, moderate, and vigorous (56); (2) PAL can be expressed as the ratio of daily energy consumption (TEE) to basal metabolism rate (BEE); and (3) The Borg scale can be utilised to attain and compare the rating of perceived exercise (RPE) (57).

Based on the classifications above and Table 4, this study systematically summarised and analysed findings from eight studies involving the effects of AVGs on objective measures of physical activity outcomes (such as EE, metabolic equivalent (MET), and HR) and PALs (such as MVPA time and percentage of MVPA time). All authors of this study independently classified the articles according to the topic and finally reached a consensus.

**Table 4:** Physical activity indicators and physical activity level.

PAL	METs	EE (kcal / min)	VO_2_max ml/(kg·min)	Max HR (%)	RPE (points)
Light intensity	<3.0	< 3.5	< 50%	< 60%	6 ~ 11
Moderate intensity	3.0 ~ 6.0	3.5 ~ 7.0	50 ~ 60%	60 ~ 75%	12 ~ 14
Vigorous intensity	> 6.0	> 7.0	> 60%	> 75%	15 ~ 20

#### 3.5.1 Effect of AVGs on physical activity

Six studies investigated the intensity of physical activity of overweight and obese college students using AVGs using different metrics. Five of these studies indicated that overweight and obese college students could achieve moderate-intensity physical activity when using AVGs (49–52, 54). Three studies indicated that AVGs could achieve moderate intensity by measuring RPE at each session (49, 51, 54). One study indicated that AVGs could reach vigorous intensity by measuring multiple metrics (%VO2max, %HRmax, and METs) (50). Another study, measured by accelerometer(% time spent in MVPA), indicated that MVPA could be increased by using AVGs with SDT function (52). Only one study indicated that AVGs provide light-intensity physical activity and minimal physiological stimulation (53). In detail, Russell et al. concluded that having gaming experience or no gaming experience or single or paired modes did not affect physiological and affective responses when using AVGs. Two studies did not investigate the intensity of physical activity when using AVGs (47, 48). In one study, although participants were presented with some moderate-intensity exercise training programmes as a reference, they were still asked to freely choose the intensity and frequency according to their preferences during the experiment (47). In the other study, physiological responses to AVGs played with only the upper and lower limbs were investigated, and the intensity of physical activity was not specified (48).

Due to the large number of types of AVGs, it was necessary to analyse the AVGs according to the aspects of choice of AVGs, mode of play, mode of exercise and control group. In terms of choice of AVGs, all five of the included studies chose to use Nintendo’s Wii Fit, a game that incorporates several different sports (e.g., tennis, boxing, skiing, and running), as their intervention method (48, 50, 51, 53, 54). Four of the studies indicated that Wii Fit could provide moderate-intensity physical activity. One study indicated that Wii Punchout could only provide minimal physiological stimulation and must be carefully considered (53). The remaining three studies used Sony’s PlayStation, Microsoft’s Xbox, and a third-person role-playing game with SDT features (47, 49, 52). Self-determination theory (SDT) is a psychological theory that aims to explain the motivations behind an individual’s behaviour, and this game based on SDT was designed to satisfy better the player’s needs for competence, autonomy, and relatedness. All three studies indicated that AVGs can positively impact physical activity.

Regarding game modes, only two studies stated whether single or paired modes were used (51, 53). However, these two studies had different results. Jacobs et al. stated that single and two-player modes could keep college students moderately physically active when using Wii Fit (51). However, Russell et al. investigated that the “Wii Punchout” game skill provided minimal physiological stimulation and was unsuitable as a replacement for traditional exercise (53). The remaining six studies did not state whether the game mode was single or paired. In terms of exercise modalities, two studies added assistive devices (stationary bikes and mini-trampolines) to the original AVGs and found that the addition of assistive devices could better utilise the AVGs to further increase physical activity levels (47, 50). One study investigated the effects of using different body parts to play the AVGs and found that using the AVGs on the upper limbs was less harmful to people with metabolic disorders (48). The remaining five studies used AVGs without additional devices. In terms of control groups, two studies investigated the effects of AVGs compared to traditional physical activity, and both showed that the use of AVGs promoted better physical activity and improved physical fitness (47, 54). The remaining six studies had no intervention (48, 51, 52) or compared to other forms of AVGs (49, 50, 53).

The majority of studies have shown positive effects of AVGs, with three studies indicating that recommended physical activity levels can be achieved with AVGs (49, 50, 54). The Wii Fit “Free Run” alone can be an alternative to traditional moderate-intensity aerobic exercise (54). Adding a mini-trampoline to the game can achieve vigorous-intensity physical activity that meets ACSM requirements (50). Structured AVGs (Nike Kinect Training) can achieve 200 kcal/session of physical activity. When used daily, it meets the energy equivalent (1,200 kcal/week) recommended by ACSM to prevent weight gain (49).

#### 3.5.2 Effect of AVGs on other factors

Seven studies investigated the positive effects of using AVGs on body composition and overall physical fitness in overweight and obese college students. Only one study concluded that AVGs were less effective (53). Of the studies that concluded that there was a positive effect, five investigated the use of AVGs to promote health-related aerobic capacities, cardiorespiratory fitness, and physical fitness, such as maximal oxygen uptake, heart rate stress multiplier, respiratory rate and vertical bounce and leg strength (47–50, 54). The remaining two highlighted that AVGs can increase MVPA time and control body weight (51, 52). In terms of exercise motivation, psychological factors and attendance, two studies explored attendance during the study period and both found that using AVGs resulted in higher attendance and thus contributed to the attainment of more significant physical activity (47, 52). Two studies indicated that using an AVG would increase motivation to exercise, which in turn would contribute to greater physical activity (50, 53). Two studies indicated that using AVGs would increase motivation to exercise, which in turn would contribute to greater physical activity. AVG increases motivation to exercise and makes overweight and obese college students more likely to want to participate in exercise (51, 52). The results of three studies involving emotions and psychology were different. In one study, an AVG based on SDT functionality increased players’ psychological need satisfaction and interest in the game (52). However, overweight and obese college students did not experience a positive effect on mood when using the Wii Punchout and Wii Fit “Free Run,” and their well-being even decreased significantly after playing “Free Run” compared to running on a treadmill (*p* = 0.018) (53, 54).

## 4 Discussions

Given the features of AVG to promote PA in overweight and obese college students, our study comprehensively reviews current research on this topic. The exercise characteristics of AVG were explored, as well as the mechanisms behind improving PA and overweight/obesity in college students. Through screening, eight RCTs met the criteria and were included in the systematic evaluation.

The majority of the included studies (seven out of eight) indicated that AVGs can have a positive impact on physical activity by modifying their habitual PA, increasing the daily time they spend engaging in PA, and ensuring their physical activity level each time they use AVGs. However, the results of one study indicated a poor effect of AVGs on physical activity (53). Differences in the choice and type of AVGs, game modes, and exercise modes can have different effects, so this systematic review focuses on analysing and discussing these points. In terms of choice and type of AVGs, the AVGs used in the seven studies were from Sony, Microsoft and Nintendo. These three manufacturers publish a wide range of AVGs and dominate almost the entire AVG market, which is the main reason why game consoles from these three manufacturers (PS, XBOX, and Wii) were commonly chosen for AVG experiments (47–51, 53, 54). Only one study used adventure AVGs developed based on the functions of SDT (52). Self-determination theory is a theory of human motivation and personality that is based on experience. Derived theory of human motivation and personality (58), which has been successfully applied in areas such as education, sports and virtual worlds (59). This AVG with SDT features not only resulted in more MVPA, but also performed well in terms of attendance, psychological satisfaction, and future motivation to play. The results of other studies have also confirmed that AVGs with SDT features can influence the satisfaction of autonomy and relatedness needs and increase players’ frequency of play (60). Theoretically designed AVGs can facilitate sedentary individuals to engage in realistic physical activities (61). Theoretical guidance is essential for physical interventions (62). Therefore, in selecting AVGs, it is necessary to consider AVGs produced by common manufacturers and choose other AVGs with theoretical guidance, especially AVGs with SDT function, which may be an ideal choice.

AVGs are different from other traditional sports training; their essence is to connect the virtual world and the natural world through playing games so that players can exercise. Different game modes and exercise methods also directly determine the effect of using AVGs. Standard game modes include single and paired modes, while exercise modes are categorised into direct use of AVGs, assistive devices, and different body parts. Two included studies described game modes, but the results varied considerably (51, 53). One study opted for Wii Fit, distinguishing between single and paired groups during the intervention. Ultimately, it was found that both single and paired groups could achieve moderate-intensity physical activity at the time of the intervention. Although the motivation to exercise was more significant in the single group than the paired group, using the Wii Fit could help college students manage weight (51). The other study’s AVG was the Wii Punchout. this study not only differentiated between single and paired groups but also between experienced and inexperienced. The results of the study showed no significant differences in peak HR, recovery HR, RPE and steps per session at the experience level (experienced and inexperienced), with the paired mode having a higher peak HR than the single-player mode. The small physiological stimulus and limited physical activity that the Wii Punchout cannot fulfil the requirements of the ACSM Exercise Guidelines (53). The difference in results between the two studies may be that using the Wii Punchout only requires some dodging and punching to play the game, which does not require much whole-body activity. In contrast, the Wii Fit has enough whole-body activity in a 45-min workout, including 30 min of aerobic exercise on the Wii and 15 min of yoga, balance, and strength activities. Other studies have also supported that more whole-body activity results in more significant energy expenditure and exercise stimulation (63). While the included studies do not have a clear answer on the effect of game mode (single or paired) on physical activity, the effect of game mode on motivation to exercise is still something to be considered in future research. Consideration of the qualities of the AVG itself and whether it provides enough whole-body activity to bring about more physiological stimulation and energy expenditure should be more of a priority.

Three studies investigated different exercise modalities. Both studies that used additional assistive equipment found that the addition of another piece of equipment to the original use of an AVG resulted in greater physical activity and better exercise outcomes (47, 50). In detail, Warburton et al. stated that the combination of an AVG and stationary bike resulted in higher attendance compared to using a stationary bike alone, and that modulation of attendance not only resulted in increased physical activity, but also improved cardiovascular metrics (maximal oxygen uptake) and health-related physical fitness (bone health, vertical spring, and leg strength) (47). Previous research has also supported the idea that higher attendance leads to greater physical activity and the amount of physical activity is essential for health (64, 65). The study by Rodrigues et al. found that physical activity intensity was increased when playing on an assistive device mini-trampoline (MT-PT) compared to performing the Wii Free Run (EXG-PT) on a hard floor (50). By % VO_2_max, HRmax, and METs measurements, the MT-PT could all be categorised as vigorous-intensity physical activity. In contrast, the EXG-PT could only achieve moderate-intensity physical activity. This result could be because playing AVG on the MT requires more muscle mobilisation to maintain balance. Therefore, the physiological stimulus is more significant, thus resulting in greater intensity of physical activity. The mini-trampoline being a promising training tool has been demonstrated in other studies; for example, the use of MT promotes balance, muscle strength, and overall health (66–68). It is worth noting that even when the Wii Free Run is used alone, previous studies have demonstrated that it can achieve moderate-intensity physical activity when used in adults, making it a more desirable choice of AVGs for physical activity interventions (69, 70). One study investigated the metabolic response to using AVGs in different parts of the body. There was more physical activity when playing with AVGs on the lower limbs, but playing with AVGs on the upper limbs only seemed better for people with heart disease. Oxygen saturation was maintained safely, whether played on the upper or lower limbs only (48). Other studies have also demonstrated that playing AVGs with only the upper limbs (e.g., the Wii’s Tennis game) can be more effortless, and therefore, physical activity is not as effective as lower limb-only or whole-body movement with AVGs (71). Some AVGs can lead to greater MVPA by themselves, such as Dance Dance Revolution (DDR) (72), Kinect Dance Central 2, and Kinect Zumba Fitness. However, the benefits of AVGs can be expanded and better utilised by adding suitable assistive devices or adopting different body parts to use AVGs according to the user’s characteristics.

In addition, both studies selected different control groups (conventional exercise and other AVGs) for comparison (49, 54). HRmax, RPE and rate pressure product (RPP) were significantly greater with Wii Fit “Free Run” than with treadmill running. Since the player can freely choose the game’s intensity, it has a great potential to not only reach moderate-intensity physical activity but even exceed traditional moderate-intensity aerobic exercise (54). A 6-week comparative study of different AVGs found that structured AVGs resulted in higher HR and EE compared to unstructured AVGs (49). Structured AVGs (Nike Kinect training) follow the principles of exercise training, increasing the intensity of the exercise to improve cardiovascular response and energy expenditure, whereas unstructured AVGs lack this capability (73). Previous research has also demonstrated the effectiveness of structured AVGs, for example, improved body fat and total cholesterol in overweight and obese adolescents through structured AVG training (74). Therefore structured AVGs are more suitable for long-term use as a physical activity intervention of choice. In terms of control group selection, many studies have shown that the advantages of AVG in increasing MVPA time, bringing more fun and promoting weight loss in sedentary college students are more obvious compared to traditional sports (75–77). However, there is still a need for more studies comparing AVGs to traditional exercise to see the benefits of AVG or to better conclude which AVGs are suitable for overweight and obese college students will be the focus of future research.

Furthermore, in terms of sample heterogeneity. Different countries do not have the exact age requirement for college students to enter school, so differences in age, gender, range of BMI, and students’ physical activity status may affect the study’s external validity. In particular, the original physical activity status of university students (e.g., active versus sedentary) can have a significant impact on experimental results, but only two of the included studies assessed university students’ status rated as low-activity (47, 52). Future studies could divide the sample more carefully to explore differences between subgroups. Regarding intervention heterogeneity, the included studies contained different types of AVGs. This intervention heterogeneity explains some of the observed differences in effects. For example, some AVGs are more challenging to provide higher-intensity physical activity, while others are more recreational and less effective in physical activity promotion (49, 53). Future studies need to delve deeper into the effects of the characteristics of different types of AVGs (e.g., game difficulty and somatic interactivity) on physical activity.

## 5 Limitations

The included studies have the following limitations. First, the study on Wii Fit for weight loss in college students had a small sample size of six participants. Moreover, one dropped out of the study, resulting in only five participants. The small sample size makes it challenging to demonstrate the effectiveness of the intervention (51).

Three studies stated limitations in the participant population (49). The AVG combined with the stationary bike study involved only males, so it is unclear whether the findings apply to females (47). The research on using MT-PTs also has some limitations. The experimental protocol only measured the acute cardiovascular response and could not evaluate the long-term impact of MT-PTs on AVG utilisation. The participants were young and healthy, so the experimental results cannot be extrapolated to other populations (50). Participants were only young men in the structured and unstructured AVG studies, which limits the generalisability of the studies. The crowd determines different sports motivations and attitudes when playing AVGs, which can lead to variations in exercise intensity and aerobic capacity. Moreover, one study only involved structured and unstructured AVGs in a single-game mode; future research must try different game combinations (49). The included studies also had some methodological limitations, such as smaller sample sizes, shorter intervention times, and shorter recovery times when using a crossover design (48, 50, 51).

## 6 Conclusion

The research in this systematic review reveals that AVGs can achieve internationally recognised and recommended physical activity intensity. AVGs combined with auxiliary equipment can better promote the physical activity of overweight and obese college students. Most studies have proposed the benefits of AVGs for physical health and physical activity. Health promotion aims to improve cardiovascular function or become a potential weight-loss tool. Promoting physical activity is mainly reflected in combating sedentary behaviour, improving the attendance rate of sports, and increasing MVPA time.

AVGs can be employed as a new sport to reflect various benefits. However, its effect on physical activity and the achievable PAL vary. Most studies have observed different results. The primary reasons for this difference are the various types of AVGs, intervention time, and equipment utilised. Different companies (such as Nintendo, Microsoft, and SONY) have distinct design intentions and audiences for each AVG. Therefore, future research should focus on the differences caused by AVG types.

The essence of an AVG is a video game that can be employed for physical exercise, so the time spent on it (attendance rate) also directly determines the exercise effect. AVGs based on SDT can improve attendance, obtain higher satisfaction of psychological needs, and lead to more MVPA time. In the future, when using AVGs to study the impact of physical activity on overweight and obese college students, we should consider using AVGs with plot and theoretical guidance design and take improving attendance rates as one of the factors to explore. The same AVG has different effects on physical activity in various sports scenes. Using AVGs with auxiliary equipment (such as an MT-PT) improves the exercise intensity, enlightening future research. Future research should explore how to overlay different external devices to achieve different physical activity effects based on using AVGs alone for intervention.

## Data availability statement

The original contributions presented in the study are included in the article/supplementary material, further inquiries can be directed to the corresponding author/s.

## Author contributions

YZ: Writing – original draft, Writing – review & editing, Software. KS: Supervision, Writing – review & editing. HS: Supervision, Writing – review & editing. CL: Supervision, Writing – review & editing. CD: Supervision, Writing – review & editing.

## Footnotes

^1^ https://www.crd.york.ac.uk/PROSPERO/

## References

[1] Ng M Fleming T Robinson M Thomson B Graetz N Margono C . Global, regional, and national prevalence of overweight and obesity in children and adults during 1980–2013: a systematic analysis for the global burden of disease study 2013. Lancet. (2014) 384:766–81. doi: 10.1016/S0140-6736(14)60460-8 24880830 PMC4624264

[2] Racette SB Deusinger SS Deusinger RH. Obesity: overview of prevalence, etiology, and treatment. Phys Ther. (2003) 83:276–88. doi: 10.1093/ptj/83.3.276 12620091

[3] Organization, W. H. (2000). Obesity: preventing and managing the global epidemic: report of a WHO consultation. Available at: https://www.google.com/books?hl=zh-CN&lr=&id=AvnqOsqv9doC&oi=fnd&pg=PA1&dq=Obesity:+Preventing+and+managing+the+global+epidemic:+report+of+a+WHO+consultation.+World+Health+Organization&ots=6XD5bsYW3P&sig=0LtdxOJobf6V8Q5bA-0zEswHZS8 11234459

[4] Dorresteijn JAN Visseren FLJ Spiering W. Mechanisms linking obesity to hypertension. Obes Rev. (2012) 13:17–26. doi: 10.1111/j.1467-789X.2011.00914.x 21831233

[5] Powell-Wiley TM Poirier P Burke LE Després J-P Gordon-Larsen P Lavie CJ . Obesity and cardiovascular disease: a scientific statement from the American Heart Association. Circulation. (2021) 143:e984–e1010. doi: 10.1161/CIR.0000000000000973, PMID: 33882682 PMC8493650

[6] Kuntz B Lampert T. Socioeconomic factors and obesity. Dtsch Arztebl Int. (2010) 107:517–22. doi: 10.3238/arztebl.2010.0517, PMID: 20737057 PMC2925342

[7] Allcott H Gentzkow M Song L. Digital addiction. Am Econ Rev. (2022) 112:2424–63. doi: 10.1257/aer.20210867

[8] Ding K Li H. Digital addiction intervention for children and adolescents: a scoping review. Int J Environ Res Public Health. (2023) 20:4777. doi: 10.3390/ijerph20064777, PMID: 36981687 PMC10049137

[9] Aziz N Nordin MJ Abdulkadir SJ Salih MMM. Digital addiction: systematic review of computer game addiction impact on adolescent physical health. Electronics. (2021) 10:996. doi: 10.3390/electronics10090996

[10] Oniz M Nazmi S Ishak G. Digital addiction and obesity in the information age: the deep connection between two modern threats and obesity education. J Exercise Sci Physical Activity Rev. (2023) 1:13–25. doi: 10.5281/ZENODO.10396706

[11] Guthold R Stevens GA Riley LM Bull FC. Worldwide trends in insufficient physical activity from 2001 to 2016: a pooled analysis of 358 population-based surveys with 1·9 million participants. Lancet Glob Health. (2018) 6:e1077–86. doi: 10.1016/S2214-109X(18)30357-7, PMID: 30193830

[12] Guthold R Stevens GA Riley LM Bull FC. Global trends in insufficient physical activity among adolescents: a pooled analysis of 298 population-based surveys with 1·6 million participants. Lancet Child & Adolescent Health. (2020) 4:23–35. doi: 10.1016/S2352-4642(19)30323-2, PMID: 31761562 PMC6919336

[13] Rey-López JP Vicente-Rodríguez G Biosca M Moreno LA. Sedentary behaviour and obesity development in children and adolescents. Nutr Metab Cardiovasc Dis. (2008) 18:242–51. doi: 10.1016/j.numecd.2007.07.008 18083016

[14] Pantenburg B Sikorski C Luppa M Schomerus G König H-H Werner P . Medical students’ attitudes towards overweight and obesity. PloS One. (2012) 7:e48113. doi: 10.1371/journal.pone.0048113, PMID: 23144850 PMC3489830

[15] Desai MN Miller WC Staples B Bravender T. Risk factors associated with overweight and obesity in college students. J Am Coll Health. (2008) 57:109–14. doi: 10.3200/JACH.57.1.109-114 18682353

[16] American College of Sports Medicine. ACSM’s guidelines for exercise testing and prescription. Lippincott Williams & Wilkins (2013). 10.1249/JSR.0b013e31829a68cf 23851406

[17] Gülü M Yagin FH Gocer I Yapici H Ayyildiz E Clemente FM . Exploring obesity, physical activity, and digital game addiction levels among adolescents: a study on machine learning-based prediction of digital game addiction. Front Psychol. (2023) 14:1097145. doi: 10.3389/fpsyg.2023.1097145, PMID: 36936011 PMC10022696

[18] Keating XD Guan J Piñero JC Bridges DM. A Meta-analysis of college students’ physical activity behaviors. J Am Coll Health. (2005) 54:116–26. doi: 10.3200/JACH.54.2.116-126 16255324

[19] Owen N Sparling PB Healy GN Dunstan DW Matthews CE. Sedentary behavior: emerging evidence for a new health risk. Mayo Clin Proc. (2010) 85:1138–41. doi: 10.4065/mcp.2010.0444 21123641 PMC2996155

[20] Foley L Maddison R. Use of active video games to increase physical activity in children: a (virtual) reality? Pediatr Exerc Sci. (2010) 22:7–20. doi: 10.1123/pes.22.1.7, PMID: 20332536

[21] Graf DL Pratt LV Hester CN Short KR. Playing active video games increases energy expenditure in children. Pediatrics. (2009) 124:534–40. doi: 10.1542/peds.2008-2851, PMID: 19596737 PMC8329994

[22] Mears D Hansen L. Active gaming: definitions, options and implementation. Strategies. (2009) 23:26–9. doi: 10.1080/08924562.2009.10590864

[23] Howe CA Barr MW Winner BC Kimble JR White JB. The physical activity energy cost of the latest active video games in young adults. J Phys Act Health. (2015) 12:171–7. doi: 10.1123/jpah.2013-0023, PMID: 24905451

[24] Monedero J Murphy EE O’Gorman DJ. Energy expenditure and affect responses to different types of active video game and exercise. PloS One. (2017) 12:e0176213. doi: 10.1371/journal.pone.0176213, PMID: 28459835 PMC5411095

[25] Grieser JD Gao Y Ransdell L Simonson S. Determining intensity levels of selected Wii fit activities in college aged individuals. Meas Phys Educ Exerc Sci. (2012) 16:135–50. doi: 10.1080/1091367X.2012.665268

[26] Wu P-T Wu W-L Chu I-H. Energy expenditure and intensity in healthy young adults during exergaming. Am J Health Behav. (2015) 39:556–61. doi: 10.5993/AJHB.39.4.12, PMID: 26018104

[27] Duncan MJ Birch S Woodfield L Hankey J. Physical activity levels during a 6-week, school-based, active Videogaming intervention using the Gamercize power stepper in British children. Med Sport. (2011) 15:81–7. doi: 10.2478/v10036-011-0014-0

[28] Majaj R. Scott T. Moran R. Kimberly D. Smith W. (2021). Physiological responses to active video games compared to treadmill walking and TV watching in obese children and adolescents. 519 10.70252/ETCL4835 PMC8136563 34055181

[29] Maddison R Straker L Palmeira A Simons M Witherspoon L. Active video games: an opportunity for enhanced learning and positive health effects? Cogn Technol. (2013) 18:6–13.

[30] Millington B. Video games and the political and cultural economies of health-entertainment In: Re-thinking leisure in a digital age. UK: Routledge (2020) 31–49.

[31] Gao Z. Fight fire with fire? Promoting physical activity and health through active video games. J Sport Health Sci. (2017) 6:1–3. doi: 10.1016/j.jshs.2016.11.009, PMID: 30356540 PMC6188911

[32] Guy S Ratzki-Leewing A Gwadry-Sridhar F. Moving beyond the stigma: systematic review of video games and their potential to combat obesity. Int J Hypertens. (2011) 2011:1–13. doi: 10.4061/2011/179124, PMID: 21629863 PMC3095884

[33] Rhodes RE Warburton DER Bredin SSD. Predicting the effect of interactive video bikes on exercise adherence: an efficacy trial. Psychol Health Med. (2009) 14:631–40. doi: 10.1080/13548500903281088, PMID: 20183536

[34] Zurita-Ortega F Chacón-Cuberos R Castro-Sánchez M Gutiérrez-Vela F González-Valero G. Effect of an intervention program based on active video games and motor games on health indicators in university students: a pilot study. Int J Environ Res Public Health. (2018) 15:1329. doi: 10.3390/ijerph15071329, PMID: 29941811 PMC6068999

[35] Han C Haichun S. Effects of active videogame and sports, play, and active recreation for kids physical education on Children’s health-related fitness and enjoyment. Games for Heal J. (2017) 6:312–318. doi: 10.1089/g4h.2017.0001 28704072

[36] Gao Z Zhang T Stodden D. Children’s physical activity levels and psychological correlates in interactive dance versus aerobic dance. J Sport Health Sci. (2013) 2:146–51. doi: 10.1016/j.jshs.2013.01.005

[37] Williams WM Ayres CG. Can active video games improve physical activity in adolescents? A review of RCT. Int J Environ Res Public Health. (2020) 17:669. doi: 10.3390/ijerph17020669, PMID: 31968642 PMC7013707

[38] Zeng N Pope Z Lee JE Gao Z. A systematic review of active video games on rehabilitative outcomes among older patients. J Sport Health Sci. (2017) 6:33–43. doi: 10.1016/j.jshs.2016.12.002, PMID: 30356538 PMC6188917

[39] McCallum S Boletsis C. Dementia games: a literature review of dementia-related serious games In: Ma M Oliveira MF Petersen S Hauge JB, editors. Serious games development and applications, vol. 8101. Berlin Heidelberg: Springer (2013). 15–27.

[40] Edwards J Jeffrey S May T Rinehart NJ Barnett LM. Does playing a sports active video game improve object control skills of children with autism spectrum disorder? J Sport Health Sci. (2017) 6:17–24. doi: 10.1016/j.jshs.2016.09.004, PMID: 30356508 PMC6188903

[41] Castro O Bennie J Vergeer I Bosselut G Biddle SJH. Correlates of sedentary behaviour in university students: a systematic review. Prev Med. (2018) 116:194–202. doi: 10.1016/j.ypmed.2018.09.016 30266213

[42] Han S-J Nagduar S Yu H-J. Digital addiction and related factors among college students. Health. (2023) 11:2943. doi: 10.3390/healthcare11222943, PMID: 37998435 PMC10671342

[43] Page MJ Moher D Bossuyt PM Boutron I Hoffmann TC Mulrow CD . PRISMA 2020 explanation and elaboration: updated guidance and exemplars for reporting systematic reviews. BMJ. (2021) 372. doi: 10.1136/bmj.n160, PMID: 33781993 PMC8005925

[44] Harriss DJ MacSween A Atkinson G. Ethical standards in sport and exercise science research: 2020 update. Int J Sports Med. (2019) 40:813–7. doi: 10.1055/a-1015-3123, PMID: 31614381

[45] Maher CG Sherrington C Herbert RD Moseley AM Elkins M. Reliability of the PEDro scale for rating quality of randomized controlled trials. Phys Ther. (2003) 83:713–21. doi: 10.1093/ptj/83.8.713 12882612

[46] De Morton NA. The PEDro scale is a valid measure of the methodological quality of clinical trials: a demographic study. Australian J Physiotherapy. (2009) 55:129–33. doi: 10.1016/S0004-9514(09)70043-1, PMID: 19463084

[47] Warburton DER Bredin SSD Horita LTL Zbogar D Scott JM Esch BTA . The health benefits of interactive video game exercise. Appl Physiol Nutr Metab. (2007) 32:655–63. doi: 10.1139/H07-038 17622279

[48] Rahayu T Aprilawati DWI Mahmud J Purwanto B Herawati L. Both upper and lower extremity-only video game-based exercises (exergaming) affect blood glucose serum levels and heart rates but not oxygen saturation in teenagers. J Physical Educ Sport. (2019) 19:802–7. doi: 10.7752/jpes.2019.s3114

[49] De Brito-Gomes JL Perrier-Melo RJ Melo De Oliveira SF De Sá Pereira Guimarães FJ Da Cunha Costa M. Physical effort, energy expenditure, and motivation in structured and unstructured active video games: a randomized controlled trial. Human Movement. (2016) 17:190–198. doi: 10.1515/humo-2016-0021

[50] Rodrigues GAA Rodrigues PC Da Silva FF Nakamura PM Higino WP De Souza RA. Mini-trampoline enhances cardiovascular responses during a stationary running exergame in adults. Biol Sport. (2018) 35:335–42. doi: 10.5114/biolsport.2018.78052, PMID: 30765918 PMC6358523

[51] Jacobs K Zhu L Dawes M Franco J Huggins A Igari C . Wii health: a preliminary study of the health and wellness benefits of Wii fit on university students. Br J Occup Ther. (2011) 74:262–8. doi: 10.4276/030802211X13074383957823

[52] Peng W Pfeiffer KA Winn B Lin J-H Suton D. A pilot randomized, controlled trial of an active video game physical activity intervention. Health Psychol. (2015) 34:1229–39. doi: 10.1037/hea0000302 26651464

[53] Russell WD Kraft JA Bergman RJ Spellman J Barnes NW. Experience level and social condition influences on heart rate, perceived exertion, and mood from interactive video game boxing. J Sport Behav. (2013) 36.

[54] Douris PC McDonald B Vespi F Kelley NC Herman L. Comparison between Nintendo Wii fit aerobics and traditional aerobic exercise in sedentary young adults. J Strength Cond Res. (2012) 26:1052–7. doi: 10.1519/JSC.0b013e31822e5967, PMID: 22446674

[55] Caspersen CJ Powell KE Christenson GM. Physical activity, exercise, and physical fitness: definitions and distinctions for health-related research. Public Health Reports (1974). (1985) 100:126–31. PMC1424733 3920711

[56] Centers for Disease Control and Prevention (CDC). School health guidelines to promote healthy eating and physical activity. MMWR Recommendations and Reports: Morbidity and Mortality Weekly Report Recommendations and Reports. (2011) 60:1–76. 21918496

[57] Borg G. A. (1962). Physical performance and perceived exertion. Available at: https://psycnet.apa.org/record/1964-00089-000

[58] Van Lange P Kruglanski A Higgins E. Handbook of theories of social psychology, *Vol.* 1. US: SAGE Publications Ltd (2012).

[59] Deci E. L. Ryan R. M. (2013). Intrinsic motivation and self-determination in human behavior. Springer Science & Business Media. Available at: https://www.google.com/books?hl=zh-CN&lr=&id=M3CpBgAAQBAJ&oi=fnd&pg=PA1&dq=Intrinsic+motivation+and+self-determination+in+human+behavior.+Springer+Science+%26+Business+Media&ots=uojHnO3YYb&sig=dxYtPum5i7gTSEuGju4iLh4yNwE

[60] Peng W Lin J-H Pfeiffer KA Winn B. Need satisfaction supportive game features as motivational determinants: an experimental study of a self-determination theory guided Exergame. Media Psychol. (2012) 15:175–96. doi: 10.1080/15213269.2012.673850

[61] Lieberman DA Chamberlin B Medina E Franklin BA Sanner BM Vafiadis DK. The power of play: innovations in getting active summit 2011: a science panel proceedings report from the American Heart Association. Circulation. (2011) 123:2507–16. doi: 10.1161/CIR.0b013e318219661d, PMID: 21518980

[62] Baranowski T Anderson C Carmack C. Mediating variable framework in physical activity interventions. Am J Prev Med. (1998) 15:266–97. doi: 10.1016/S0749-3797(98)00080-4 9838973

[63] Graves LEF Ridgers ND Stratton G. The contribution of upper limb and total body movement to adolescents’ energy expenditure whilst playing Nintendo Wii. Eur J Appl Physiol. (2008) 104:617–23. doi: 10.1007/s00421-008-0813-8, PMID: 18607619

[64] Warburton DER. Health benefits of physical activity: the evidence. Can Med Assoc J. (2006) 174:801–9. doi: 10.1503/cmaj.051351, PMID: 16534088 PMC1402378

[65] Warburton DER Sheel AW Hodges ANH Stewart IB Yoshida EM Levy RD . Effects of upper extremity exercise training on peak aerobic and anaerobic fitness in patients after transplantation. Am J Cardiol. (2004) 93:939–43. doi: 10.1016/j.amjcard.2003.12.030, PMID: 15050506

[66] Karakollukçu M Aslan CS Paoli A Bianco A Sahin FN. Effects of mini trampoline exercise on male gymnasts’ physiological parameters: a pilot study. J Sports Med Phys Fitness. (2015) 55:730–4. 24921617

[67] Sukkeaw W Kritpet T Bunyaratavej N. A comparison between the effects of aerobic dance training on Mini-trampoline and hard wooden surface on bone resorption, Health-Related Physical Fitness, Balance, and Foot Plantar Pressure in Thai Working Women (2015). 98, S58–S64. 26529816

[68] Working Group of Gender Cardiovascular Disease of the Italian Society of Cardiology Cugusi L Manca A Serpe R Romita G Bergamin M . Effects of a mini-trampoline rebounding exercise program on functional parameters, body composition and quality of life in overweight women. J Sports Med Phys Fitness. (2018) 58:287–294. doi: 10.23736/S0022-4707.16.06588-9, 27441918

[69] Guderian B Borreson LA Sletten LE Cable K Stecker TP Probst MA . The cardiovascular and metabolic responses to Wii fit video game playing in middle-aged and older adults. J Sports Med Phys Fitness. (2010) 50:436–42. PMID: 21178930

[70] Miyachi M Yamamoto K Ohkawara K Tanaka S. METs in adults while playing active video games: a metabolic chamber study. Med Sci Sports Exerc. (2010) 42:1149–53. doi: 10.1249/MSS.0b013e3181c51c78 19997034

[71] Peng W Lin J-H Crouse J. Is playing Exergames really exercising? A Meta-analysis of energy expenditure in active video games. Cyberpsychol Behav Soc Netw. (2011) 14:681–8. doi: 10.1089/cyber.2010.0578, PMID: 21668370

[72] Maloney AE Bethea TC Kelsey KS Marks JT Paez S Rosenberg AM . A pilot of a video game (DDR) to promote physical activity and decrease sedentary screen time. Obesity. (2008) 16:2074–80. doi: 10.1038/oby.2008.295, PMID: 19186332

[73] Swain DP Franklin BA. Comparison of cardioprotective benefits of vigorous versus moderate intensity aerobic exercise. Am J Cardiol. (2006) 97:141–7. doi: 10.1016/j.amjcard.2005.07.130, PMID: 16377300

[74] Adamo KB Rutherford JA Goldfield GS. Effects of interactive video game cycling on overweight and obese adolescent health. Appl Physiol Nutr Metab. (2010) 35:805–15. doi: 10.1139/H10-078, PMID: 21164552

[75] McDonough D Pope Z Zeng N Lee J Gao Z. Comparison of college students’ energy expenditure, physical activity, and enjoyment during exergaming and traditional exercise. J Clin Med. (2018) 7:433. doi: 10.3390/jcm7110433, PMID: 30423805 PMC6262538

[76] Sween J Wallington SF Sheppard V Taylor T Llanos AA Adams-Campbell LL. The role of exergaming in improving physical activity: a review. J Phys Act Health. (2014) 11:864–70. doi: 10.1123/jpah.2011-0425, PMID: 25078529 PMC4180490

[77] Yang C Wickert Z Roedel S Berg A Rothbauer A Johnson M . Time spent in MVPA during exergaming with Xbox Kinect in sedentary college students. Int J Exerc Sci. (2014) 7:4.

